# Physiological acclimation of elk during population restoration in the Missouri Ozarks, USA

**DOI:** 10.1093/conphys/coac009

**Published:** 2022-03-04

**Authors:** Ellen M Pero, M Colter Chitwood, Aaron M Hildreth, Barbara J Keller, Rami J Millspaugh, Jason A Sumners, Lonnie P Hansen, Jason L Isabelle, Creagh W Breuner, Joshua J Millspaugh

**Affiliations:** 1 Wildlife Biology Program, University of Montana, 32 Campus Drive, Missoula, MT 59812, USA; 2 Natural Resource Ecology & Management, Oklahoma State University, 008C Agriculture Hall, Stillwater, OK 74078, USA; 3 Missouri Department of Conservation, 3500 E Gans Rd, Columbia, MO 65201, USA; 4 Minnesota Department of Natural Resources, 500 Lafayette Rd, St. Paul, MN 55155, USA; 5 School of Natural Resources, University of Missouri, Columbia, MO 65211, USA; 6 Missouri Department of Conservation, 2901 W Truman Blvd, Jefferson City, MO 65102, USA; 7 Division of Biological Sciences, University of Montana, 32 Campus Drive, Missoula, MT 59812, USA

## Abstract

Conservation translocations—the intentional movement of animals to restore populations—have increased over the past 30 years to halt and reverse species declines and losses. However, there are many challenges translocated animals face that should be considered for restoration programs to be successful. Understanding how long it takes for translocated animals to acclimate to these challenges and their new landscape is a critical component of post-release population management. Physiological measures such as hormone responses are increasingly used to assess animal responses and acclimation to disturbances including translocation. We determined the physiological acclimation period of elk (*Cervus canadensis*) translocated to the Missouri Ozarks, USA, as part of a restoration effort. From 2011 to 2013, we translocated 108 GPS-radio-collared elk from Kentucky, USA, to Missouri, USA, and collected faecal samples for glucocorticoid metabolite extraction to use as an indicator of physiological acclimation. We modelled the response of population-wide faecal glucocorticoid metabolites (fGCMs) across the initial 9 years of the restoration in response to days following release and additional site-specific covariates. Presence of white-tailed deer (*Odocoileus virginianus*) hunts and monthly precipitation levels were positively and negatively associated with fGCM levels, respectively. Concurrent with influences from site-specific conditions on the release landscape, fGCM levels declined following release. We identified a breakpoint in fGCM decline at ~42 days following translocation releases suggesting elk acclimated physiologically relatively quickly compared to other species. The fast physiological acclimation by Missouri elk suggests effective use of temporary post-release management efforts. Determining how quickly animals acclimate following translocations allows researchers to tailor post-release management plans to each species’ needs, thus maximizing the success of future translocation efforts while minimizing costs.

## Teaser

Wildlife translocation is an important, but costly and challenging, conservation tool. Understanding how long it takes wildlife to acclimate to new landscapes improves post-release management. We used fecal glucocorticoid metabolites to document a relatively brief physiological acclimation period in elk following translocation. We propose applications to species-specific translocation planning.

## Introduction

Biological communities are experiencing declines worldwide in what has been called the ‘sixth great extinction’ (Ceballos *et al.,* 2017). Terrestrial communities have lost over 20% of their original biodiversity globally and three quarters of large land mammals have been extirpated from their original ranges (Diaz *et al.,* 2019). Conservation translocation—the intentional movement of animals to restore populations (IUCN, 2013)—has emerged over the past 30 years as an important conservation tool to halt and reverse species declines. Nearly 700 reintroduction-based translocation efforts occurred in the USA alone by 1989 (Griffith *et al.,* 1989) and the number has subsequently increased (Seddon and Armstrong, 2016). Despite increases in the practice, translocation projects have been plagued by failures often attributed to unavoidable challenges and disruptions to translocated individuals (Griffith *et al.,* 1989; Teixeira *et al.,* 2007).

Wildlife experience challenges associated with the translocation process during their acclimation to the new landscape (Dickens *et al.,* 2010; Teixeira *et al.,* 2007). For example, during translocation, animals often experience multiple captures, periods of captivity and/or quarantine, disease testing and intervention, containment and transfer and release into foreign systems with novel pressures (Dickens *et al.,* 2010). This series of successive translocation challenges represents a prolonged exposure to stress and is one of the biggest threats to restoration success (Armstrong *et al.,* 2017; Dickens *et al.,* 2010; Teixeira *et al.,* 2007). If translocated wildlife are unable to adequately respond to prolonged challenges through behavioural and physiological modifications, animals risk physiological disruption (Romero *et al.,* 2009). Physiological disruptions in turn make animals more susceptible to increased mortality and reproductive failure when acclimating to their new landscape and these post-release effects can determine whether a translocation is successful (Armstrong and Reynolds, 2012).

Post-release effects can be mitigated through management interventions (Harrington *et al.,* 2013). In particular, managers can provide supplemental food (Castro *et al.,* 2003) or protection from predators (Villemey *et al*., 2013) during the acclimation period. Managers may also choose to limit the amount of human viewing or recreation opportunities available to the public while a population acclimates to minimize additional challenges to translocated populations. For example, managers closed trapping seasons within a 625-km^2^ area to protect a recently translocated fisher population (*Martes pennanti*) in southwestern Oregon (Aubry and Lewis, 2003). However, such management actions are expensive and sometime controversial (Coz *et al*., 2020). Understanding how long provisions or protections need to be applied following a translocation effort can maximize time and cost efficiency (Moehrenschlager and Lloyd, 2016). For this reason, knowing the length of time necessary for a population to acclimate to its new landscape can inform post-release management and is important to translocation success.

With recent attention on population acclimation, it is thought that duration of time required to reach acclimation following translocation varies among species (Armstrong *et al.,* 2017); however, species-specific data on acclimation duration is limited (Franceschini *et al.,* 2008, Jachowski *et al*., 2013b; Ji *et al.,* 2013; Yang *et al.,* 2019). Understanding the time to acclimation and variation across species could help determine species-specific sensitivities to post-release effects and how reactive species are to translocation challenges. Base knowledge of species-specific sensitivities to translocation challenges may ultimately assist biologists in planning future translocation efforts. Understanding the spectrum of translocation sensitivities across species is also necessary to inform species- or taxa-specific translocation guidelines recommended by the IUCN (IUCN, 2013).

Previous investigations on acclimation have focused on estimating duration through changes in survival (Armstrong *et al.,* 2017), but the demographic data required is resource intensive (e.g. mark-recapture studies) and does not reflect finer-scale impacts. Moreover, because mortality is ostensibly the coarsest metric to gather, mangers may benefit from finer-scale bioindicators of acclimation that may be useful in forecasting ultimate demographic trends. Measuring the behavioural or physiological acclimation of wildlife may provide more sensitive response metrics to translocation that may provide increased mechanistic understanding and forecasting of ultimate population trends (Wikelski and Cooke, 2006). Glucocorticoid hormones (GCs) are highly conserved steroid hormones that, in addition to metabolic regulation, modulate and, in turn, reflect physiological and behavioural responses to environmental challenges (McEwen and Wingfield, 2003). GCs secreted into the blood are metabolized and present in multiple non-plasma materials that can be collected frequently and noninvasively to reflect integrated GC levels over tissue- and species-specific excretion intervals (Dantzer *et al.,* 2014). Faecal glucocorticoid metabolites (fGCMs) are one non-plasma material commonly used when sampling plasma is not preferred or possible (Palme, 2019). Researchers increasingly use GCs as sensitive physiological markers of individual and population response to translocation (Dickens *et al.,* 2010; Teixeira *et al.,* 2007) and commonly observe elevations in GCs following release (Franceschini *et al.,* 2008; Jachowski *et al*., 2013b). As such, the return of GC levels to baseline may be used to indicate physiological acclimation following translocation.

Although GCs and their metabolites are commonly used to indicate responses to translocation challenges, they are less commonly used to understand the duration of acclimation and, in turn, inform the sensitivity of species to translocation-related conservation actions. To bridge this information gap, we use fGCMs as an indicator of acclimation status in a translocated elk (*Cervus canadensis*) population in Missouri, USA. Evidence suggests elk acclimate well to different forms of disturbance (Van Dyke *et al.,* 2012) to the point that concern exists for high levels of elk habituation in unhunted populations (Thompson and Henderson, 1998). Further, increasing evidence associates underlying GC physiology with animal movement behaviour (Jachowski *et al*., 2013a, 2018; Jachowski and Singh, 2015), and initial investigation into the movements made by elk translocated to Missouri suggested little behavioural disruption following release (Bleisch *et al.,* 2017). We hypothesized that the recently translocated Missouri elk population would similarly show little physiological sensitivity to translocation by demonstrating a relatively fast period of fGCM acclimation. In addition to estimating the physiological acclimation period for Missouri elk, we compared our results to durations for other species to consider a broader species-specific spectrum of translocation sensitivity. A better understanding of species-specific sensitivities to translocation will ultimately inform species-specific translocation protocols as advocated by the IUCN to improve conservation efforts (IUCN, 2013).

## Methods

### Animal translocations

We translocated 108 elk from eastern Kentucky, USA, to the southeastern Missouri Ozarks, USA (91°24′ to 90°58′W and 37°0′ to 37°19′N; Bleisch *et al.,* 2017) in three successive cohorts from 2011 to 2013. The nearest neighbouring restored elk population was in Arkansas and separated from the Missouri elk range by ~250 mi (Dent *et al.,* 2012). We captured elk from the source population in January of each year (2011–2013) and held them in quarantine corral facilities at the capture site for 102–129 days before overnight trailer transport to Missouri to perform health testing. Upon arrival in Missouri, and prior to release, we held elk for an additional quarantine period of 19–34 days in outdoor holding corrals at Peck Ranch Conservation Area, which is managed by the Missouri Department of Conservation (MDC).

We released elk in June of each year (2011: June 1; 2012: June 19 and June 23; 2013: June 7). Two elk died prior to releases (Chitwood *et al.,* 2018), and the demographic composition of release cohorts differed in each year: 2011 (*n* = 34), 15 adult females (2+ y), 5 yearling females, 6 two-year-old males, 8 yearling males; 2012 (*n* = 33), 22 adult females, 3 yearling females, 4 two-year-old males, 4 yearling males; and 2013 (*n* = 39), 20 adult females, 16 yearling females, 3 yearling males. Prior to release, we fit all elk with GPS-VHF collars (RASSL custom 3D cell collar, North Star Science and Technology, King George, VA, or G2110E Iridium-GPS series model, Advanced Telemetry Systems, Isanti, Minnesota, USA) and affixed passive integrated transponder (PIT) and ear tags.

### Sample collection

We collected fresh faecal samples with semi-regular frequency (from September 2011 to December 2014 and from January 2018 to November 2019) without observation or knowledge of individual elk identity. We randomized collection of elk faecal samples across the landscape by randomly selecting GPS-collared elk IDs and collecting a fresh faecal sample from the area of their most recent location within the previous 6 h. While this approach did not allow us to ascribe individual identity to collected samples, it did ensure random sampling across the release landscape to which elk demonstrated relatively high site fidelity (Bleisch *et al.,* 2017). Previous studies found little difference in fGCM estimates between anonymous and individual based collection approaches in ungulate species (Corlatti, 2018; Huber *et al.,* 2003).

Upon sample collection, we randomly subsampled 5–10 faecal pellets from pellet groups that appeared fresh. We avoided collecting samples after rain events to preserve the integrity of the fGCMs within faecal samples (Washburn and Millspaugh, 2002) and facilitate confidence around recency of pellet deposition. We homogenized pellets with a mallet prior to storage within a −20^°^C freezer until assay preparation (Millspaugh and Washburn, 2003).

**Table 1:** Covariate table including covariate name, description and possible values for three categories of variables hypothesized to explain fGCM variation in the restored Missouri elk (*C. canadensis*) population

Category	Covariate	Description	Values
Translocation	Proportion translocated	Proportion of population translocated in year	1, 0.5, 0.33, 0
	Restoration year	Year of restoration effort	1–9
	Days from release	Number of days following most recent translocation release	1–2357 days
Climate	Daily precipitation	Average precipitation from previous day in alignment with fGCM passage time for elk (Wasser *et al.,* 2000)	0–5.99 (cm)
	Daily temperature	Average temperature from previous day in alignment with fGCM passage time for elk (Wasser *et al.,* 2000)	−12.31 to 29.44 (°C)
	Monthly precipitation	Average precipitation across month	1.68–23.87 (cm)
	Monthly temperature	Average temperature across month	−2.76 to 27.80 (°C)
Disturbance	3-day hunt window	Occurrence of deer (*O. virginianus*) hunt in area within 3-day window	Yes/no
	5-day hunt window	Occurrence of deer hunt in area within 5-day window	Yes/no
	10-day hunt window	Occurrence of deer hunt in area within 10-day window	Yes/no
	3-day hunt-type window	Occurrence and type of deer hunt in area within 3-day window	None, archery, rifle, muzzleloader
	5-day hunt-type window	Occurrence and type of deer hunt in area within 5-day window	None, archery, rifle, muzzleloader
	10-day hunt-type window	Occurrence and type of deer hunt in area within 10-day window	None, archery, rifle, muzzleloader

### Sample preparation and assay

We followed established protocols for fGCM extraction, dilution and assay outlined by Wasser *et al.* (2000) and physiologically validated for elk (Millspaugh *et al.,* 2001). Briefly, we freeze-dried samples then ground and sifted them through stainless steel mesh for thorough mixing. We subsampled dried and sifted faeces to a standardized weight of ~0.2 g for each sample. We extracted metabolites by washing dried faeces in 2.0 ml 90% methanol, vortexing for 30 min and centrifuging for 20 min at 4°C. We stored the resulting supernatant in a −20°C freezer until assayed. We used corticosterone I125 radioimmunoassay kits (MP Biomedicals, Solon, OH) and followed MP Biomedical assay protocol except for halving reagent volumes (Millspaugh *et al.,* 2001).

We assayed a first batch of samples collected in 2011–2014 (*N* = 935) in a randomized order in 2014 over 12 assays. Average inter-assay variation for 2011–14 assays was 2.92% and intra-assay variation was 1.51%. We assayed a second batch of samples collected in 2018 and 2019 (*N* = 236) together in a randomized order in 2020 over six assays. Average inter-assay variation for 2018–19 assays was 6.99% and intra-assay variation was 1.63%. We duplicated the assay of 50 freeze-dried faecal samples collected in 2011–2014 at the time of assay for the 2018 and 2019 samples to test for bias between batches. We stratified selection of the duplicated samples across low [*N* = 18; 0–20 ng/g], medium [*N* = 14; 21–50 ng/g] and high [*N* = 18; 51–200 ng/g] fGCM values. Samples were highly correlated (Pearson’s r = 0.95), and we did not detect any difference in fGCM values between batches that was beyond a consistent, marginal decline expected with extended storage (6–9 years) of lyophilized samples in a −20°C freezer (paired-samples *t*-test: *t* = −6, *P* < 0.05, mean difference [95% CI] = −7.27 [−9.78—4.77]).

### Statistical analyses

We modelled the dynamics of elk fGCM responses to translocation with a two-step process. First, we built a generalized linear model to draw inferences on fGCM responses relative to the effect of translocation along with other covariates hypothesized to influence elk fGCMs. We then performed a breakpoint analysis (Muggeo, 2003) on the model to identify when physiological acclimation occurred as evidenced by a significant change in the slope of fGCM response in the days following translocation releases. Because we were unable to collect pre-translocation faecal samples to determine within-population baseline fGCM values, we relied on comparison to reference values from established elk populations that were determined using the same laboratory methodology and reported elsewhere in the literature (Washington: Jachowski *et al.,* 2015; South Dakota: Millspaugh *et al.,* 2001).

To build the generalized linear model for the first step in our analysis, we considered covariates in three categories hypothesized to challenge elk: translocation factors, climate and human disturbance (Table 1). Translocation covariates included days from most recent translocation release, year of restoration and the proportion of animals released within the year (Table 1). Climate covariates included temperature and precipitation covariates averaged over the month and previous day to reflect potential thermoregulatory and/or drought challenges (Romero, 2002; Table 1). We used measures from the previous day for daily averages of climatic variables to align with the GCM excretion profile of elk (Wasse *et al.,* 2000). Human disturbance covariates included factors related to the occurrence and type (gun, bow, muzzleloader) of managed white-tailed deer (*Odocoileus virginianus*) hunts that took place sporadically October–December within Peck Ranch Conservation Area. Hunting is a major challenge to target animals (Santos *et al.,* 2018). Although elk were not hunted, we included these human disturbance covariates to reflect potential challenges associated with human activity and use of firearms on the landscape. As we were unsure the duration of potential challenge following the end of the managed deer hunts, we compared models reflecting a 3-, 5- and 10-day period wherein faecal samples were considered to be within the hunting window.

Within each category of covariates, we fit models with each covariate separately in program R using the ‘stats’ package (R Core Team, 2021) and added two additional variables reflecting day-of-year terms (Eqs. 1 and 2: Jammalamadaka and Lund, 2006; Table 2).

[Eq. 1] \begin{align*} \mathrm{Sine\ day\ of\ year} =\mathrm{sine}\Big(\frac{2\pi[ day\ of\ year]}{365}\Big). \end{align*}

[Eq. 2] \begin{align*} \mathrm{Cosine\ day\ of\ year} = \mathrm{cosine}\Big(\frac{2\pi [ day\ of\ year]}{365}\Big). \end{align*}

**Table 2:** Model table including model descriptions and model structures for each of three categories of variables hypothesized to explain fGCM variation in the restored Missouri elk (*C. canadensis*) population

Category	Model description	Model structure
Translocation	Null	fGCM ~ 1
	Day of year	fGCM ~ sin.day + cos.day
	Day of year + proportion translocated in year	fGCM ~ sin.day + cos.day + prop.trans
	Day of year + days from most recent release	fGCM ~ sin.day + cos.day + df.release
	Day of year + year of restoration	fGCM ~ sin.day + cos.day + restor.yr
Climate	Null	fGCM ~ 1
	Day of year	fGCM ~ sin.day + cos.day
	Day of year + avg daily precipitation	fGCM ~ sin.day + cos.day + d.prcp
	Day of year + avg daily temperature	fGCM ~ sin.day + cos.day + d.temp
	Day of year + avg monthly precipitation	fGCM ~ sin.day + cos.day + m.prcp
	Day of year + avg monthly temperature	fGCM ~ sin.day + cos.day + m.temp
Disturbance	Null	fGCM ~ 1
	Day of year	fGCM ~ sin.day + cos.day
	Day of year + deer hunt in 3-day window	fGCM ~ sin.day + cos.day + hunt.3d
	Day of year + deer hunt in 5-day window	fGCM ~ sin.day + cos.day + hunt.5d
	Day of year + deer hunt in 10-day window	fGCM ~ sin.day + cos.day + hunt.10d
	Day of year + deer hunt type in 3-day window	fGCM ~ sin.day + cos.day + huntyp.3d
	Day of year + deer hunt type in 5-day window	fGCM ~ sin.day + cos.day + huntyp.5d
	Day of year + deer hunt type in 10-day window	fGCM ~ sin.day + cos.day + huntyp.10d

We included these day-of-year terms across all models to control for the strong seasonal rhythms of fGCMs (Romero, 2002). We compared support for each model within these three categories using Akaike Information Criterion for small sample sizes (AICc; Burnham and Anderson, 2002) in program R using the ‘performance’ package (Lüdecke *et al.,* 2021).

We based inference on a model which combined the most supported model within each covariate category. To address model uncertainty, we retained all covariates from models that were within 2 AICc units of the most supported model within each of the three categories to the combined model. If supported covariates showed multicollinearity (defined as VIFs > 5: Thompson *et al.,* 2017), we selected covariates from only the most supported model in that category for the combined model. We examined normality assumptions and model fit using the R package ‘performance’ (Lüdecke *et al.,* 2021).

For the second step of our analysis, we assessed fGCM acclimation using piecewise linear regression to test for the occurrence of a breakpoint at which the regression curve from the combined model characterizing fGCMs changed its slope relative to the explanatory variable of ‘days from release’ (package ‘segmented’; Muggeo, 2008). Convergence of the algorithm from the function ‘segmented’ demonstrates the existence of a breakpoint and a change in the linear relationship within the regression model (Muggeo, 2003).

## Results

We collected and assayed a total of 1171 elk faecal samples from 2011 to 2019. Days from release, average monthly precipitation and average daily temperature and occurrence and/or type of deer hunt within 10-day or 5-day intervals were most supported within translocation, climate and disturbance categories, respectively (Table 3). Within the disturbance category, the three top models reflecting occurrence of a deer hunt within 10-day and 5-day intervals and the model reflecting both occurrence and type of hunt within a 10-day interval (gun, bow, muzzleloader, no hunt) were within 2 AIC units of each other (Table 3). Because these three hunting covariates were highly correlated, we only included the covariates from the lowest AIC model reflecting occurrence of a hunt within a 10-day interval into the global model (Eq. 3):

[Eq. 3] \begin{align*} fGCM &= sin.day+cos.day+m.precip+d.temp+hunt.\textit{10}d\nonumber\\& \quad+ df.release \end{align*}

**Table 3:** Model selection results for three categories of variables hypothesized to explain fGCM variation in the restored Missouri elk (*C. canadensis*) population

Category	Model	K	ΔAICc	LL	w_i_
Translocation	fGCM ~ sin.day + cos.day + df.release	5	0	−4544.97	0.91
	fGCM ~ sin.day + cos.day + restor.yr	5	4.53	−4547.23	0.09
	fGCM ~ sin.day + cos.day + prop.trans	5	17.71	−4553.82	0.00
	fGCM ~ sin.day + cos.day	4	30.59	−4561.27	0.00
	fGCM ~ 1 (intercept only)	2	168.82	−4632.40	0.00
Climate	fGCM ~ sin.day + cos.day + m.prcp	5	0	−4557.92	0.58
	fGCM ~ sin.day + cos.day + d.temp	5	1.84	−4558.84	0.23
	fGCM ~ sin.day + cos.day + d.prcp	5	3.71	−4559.77	0.09
	fGCM ~ sin.day + cos.day	4	4.68	−4561.27	0.06
	fGCM ~ sin.day + cos.day + m.temp	5	5.23	−4560.53	0.04
	fGCM ~ 1 (intercept only)	2	142.92	−4632.40	0.00
Disturbance	fGCM ~ sin.day + cos.day + hunt.10d	5	0	−4556.47	0.34
	fGCM ~ sin.day + cos.day + hunt.5d	5	0.48	−4556.71	0.27
	fGCM ~ sin.day + cos.day + huntyp.10d	7	1.28	−4555.09	0.18
	fGCM ~ sin.day + cos.day + hunt.3d	5	2.63	−4557.79	0.09
	fGCM ~ sin.day + cos.day + huntyp.5d	7	3.46	−4556.18	0.06
	fGCM ~ sin.day + cos.day + huntyp.3d	7	4.28	−4556.59	0.04
	fGCM ~ sin.day + cos.day	4	7.57	−4561.27	0.01
	fGCM ~ 1 (intercept only)	2	145.80	−4632.4	0.00

We report number of parameters (K), difference AIC corrected for small sample sizes from the most supported model (ΔAICc), log likelihood (LL) and the Akaike weights (w_i_) for each model.

**Figure 1:**
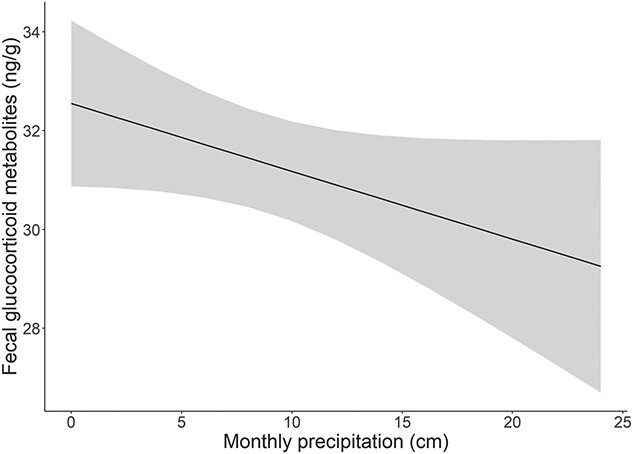
Predicted effect of average monthly precipitation on fGCM response in the restored Missouri elk (*C. canadensis*) population in the initial 9 years of restoration (2011–2019).

**Figure 2:**
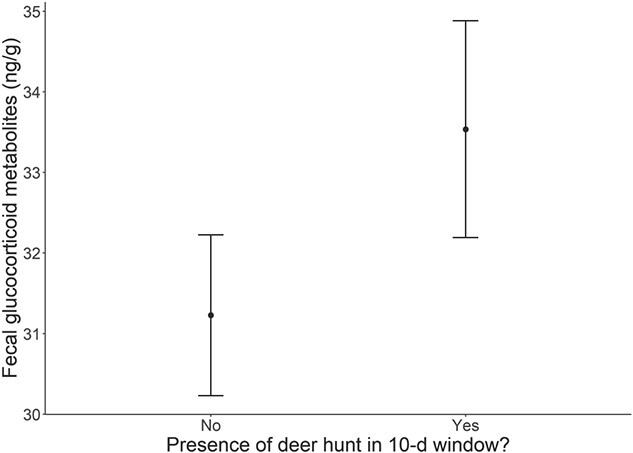
Predicted effect of the presence (no/yes) of a white-tailed deer (*O. virginianus*) hunt within a 10-day window on fGCM response in the restored Missouri elk (*C. canadensis*) population in the initial 9 years of restoration (2011–2019).

Results from the final regression model indicated that fGCMs decreased with number of days following release (β = −0.0024, SE = 0.0005, *P* < 0.001). Higher average monthly precipitation was marginally associated with lower fGCMs (β = −0.1372, SE = 0.0789, *P* < 0.1; Fig. 1), while average daily temperature had no significant relationship with fGCMs (β = 0.0676, SE = 0.0761, *P* > 0.1). Higher fGCMs were associated with the occurrence of a deer hunt within a 10-day interval (β = 2.3082; SE = 0.8678, *P* < 0.01; Fig. 2). Circular day of year covariates were strongly associated with fGCMs (sin.day: β = −2.2413, SE = 0.6449, *P* < 0.001; cos.day: β = −7.1297, SE = 1.1311, *P* = 0.001).

**Figure 3:**
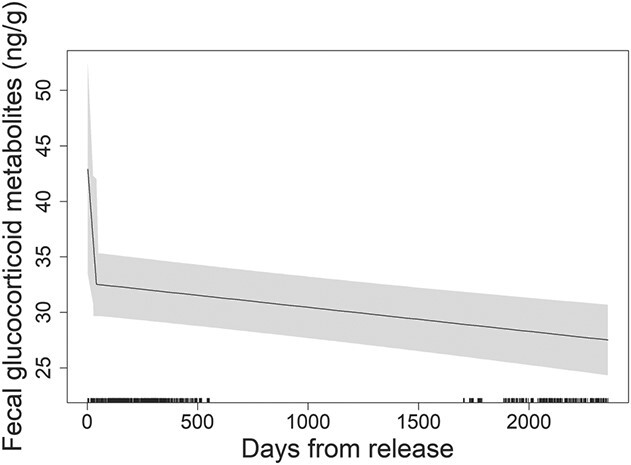
Predicted effect of days from the most recent translocation release on fGCM response in the restored Missouri elk (*C. canadensis*) population in the initial 9 years of restoration (2011–2019), with estimated breakpoint and indication of physiological acclimation occurring at ~42 days. Rugs indicate sampling occurrences.

Segmented analysis detected a breakpoint in fGCM values at 41.99 days following release, with a decrease of ~10 ng/g immediately before and after the predicted breakpoint (~27% difference; Fig. 3). The effect of days from release continued to be negative after 42 days, suggesting elk showed continued adjustment to their landscape following the initial indication of acclimation at 42 days. However, the size of negative effect was marginal relative to before the breakpoint (days from release before breakpoint: β = −0.2657, SE = 0.2030; days from release after breakpoint: β = −0.0065, SE = 0.0020), indicating minimal continued acclimation of fGCMs.

## Discussion

Glucocorticoid hormones regulate and reflect physiological responses to environmental challenges (McEwen and Wingfield, 2003), and animals typically respond to the challenge of translocation with elevated levels of GCs (Dickens *et al.,* 2010). The duration of elevated GC levels is not well described across species but has implications for post-release management and ultimate success of translocation. We observed a breakpoint in the decline of fGCMs after 42 days post-release, reflecting a relatively fast population-level acclimation period by elk to the Missouri Ozark landscape. The ~27% difference in fGCM before and after the breakpoint is below the 2-fold differences observed between elk populations in high- and low-disturbance sites in Washington (Jachowski *et al.,* 2015), and between high-human disturbance summer periods and lower-disturbance, winter periods for an elk population in South Dakota (Millspaugh *et al.,* 2001). However, because we took a population-level approach, the effect of days from release was likely diluted across years as proportionally less of the population was actively released during the second and third translocation years. Thus, lower fGCMs from animals translocated in previous years would dilute the observed response, making the decrease in fGCMs after release more gradual for years two and three after translocation. However, we still observed a significant decline in fGCMs and a breakpoint at the first 42 days following release of animals across all years, which suggests a strong effect.

While we observed an approximate 42-day physiological acclimation period in Missouri elk, comparisons among species with available data suggest there is considerable variation in acclimation duration (Dickens *et al.,* 2010; Jachowski *et al*., 2013b). Such variability in acclimation periods indicates there is likely a wide spectrum of variation in species sensitivity to translocation. For example, researchers detected elevated fGCM levels 20 years after translocation in African elephants (*Loxodonta africana*) (Jachowski *et al*., 2013b). The greater sensitivity to translocation observed in elephants suggested by the long-term physiological acclimation may be expected for a species with strong and complex social systems (Wittemyer *et al*., 2005), long memories and advanced cognitive capacities (Byrne *et al.,* 2009). Conversely, captively bred Przewalski’s horses (*Equus ferus przewalskii*) appear to be relatively insensitive to translocation challenges, indicated by physiological acclimation within 72 h of release (Ji *et al.,* 2013). The fast acclimation observed for Przewalski’s horses may be attributed to generations of captive breeding (Ji *et al.,* 2013); however, which species-specific traits contribute to variation in sensitivities to translocation remains an open area of investigation. Together with white rhinoceroses (*Ceratotherium simum*: 32 days; Yang *et al.,* 2019) and Grevy’s zebras (*Equus grevyi*: 11–18 weeks; Franceschini *et al.,* 2008), the physiological acclimation period of elk falls between the long-term duration of African elephants and the near immediate response by Przewalski’s horses. There are myriad additional factors that may influence a population’s response to translocation, including number, intensity and duration of challenges associated with translocation and the release landscapes (Dickens *et al.,* 2010; Romero and Wingfield, 2015). Species-specific sensitivity may thus be most appropriately used to form baseline expectations for anticipating species-specific population response to translocation and informing post-release management plans.

Additional context-specific factors should be considered as potentially influencing a population’s acclimation period. For example, the relatively fast acclimation of the restored Missouri elk population could have been affected by lactation status of females as calves moved from nursing to foraging; however, calving dates in Missouri were wide-ranging over the restoration (Keller *et al.,* 2015), making it unlikely that lactation status could drive the response we saw in the breakpoint analysis. Likewise, there are documented seasonal patterns of declining fGCMs from summer to fall (Millspaugh *et al.,* 2001), but such a seasonal pattern does not align with the distinct breakpoint we detected. Given our attempt to control for such potential effects via day of year terms, it seems more likely that in addition to underlying species-specific sensitivity, fast acclimation may have been facilitated by post-release management intended to assist acclimation. The MDC bolstered forage resources through planting of high-quality food plots, limited human disturbance by restricting public elk-viewing opportunities during calving, prohibited elk hunting on the recently restored population and chose a release site with relatively low levels of human development and a reduced predator guild (Dent *et al.,* 2012). While durations of physiological acclimation are unknown for other translocated elk populations, comparisons of movement patterns between the restored Missouri population and a restored Ontario population receiving less post-release intervention may suggest indication of faster behavioural acclimation in the Missouri population (Ontario: 1–3 years, Fryxell *et al.,* 2008; Missouri: < 6 months, Bleisch *et al.,* 2017).

The relatively fast physiological acclimation in the Missouri population was discernable despite subsequent climatic and human disturbance stressors occurring on the release landscape. For example, human disturbance is known to be a primary challenge influencing fGCM response in established elk populations (Jachowski *et al.,* 2015; Millspaugh *et al.,* 2001). While we did observe a small effect of increased fGCMs associated with hunting activity associated with managed deer hunts, the timing of a breakpoint in fGCM decline prior to hunts suggests a fast physiological acclimation to the challenge of translocation that was earlier and more influential than any subsequent effects of deer hunting or climatic variability (e.g. precipitation) on the Missouri landscape.

Understanding the duration of acclimation can inform the length of time that post-release management activities intended to facilitate acclimation are necessary. For example, the MDC maintained restrictions on public elk-viewing opportunities annually within the core elk range during the calving season until 2017 (3 years after final release of elk). The rapid acclimation in fGCMs we observed following translocation supports the benefits of public-viewing restrictions in the initial months following releases but suggests such restrictions may not be necessary over subsequent years. Conversely, our finding of a persisting decline, though minimal, in fGCMs after the signal of acclimation suggests elk may continue to adjust to their landscape beyond the primary period of initial physiological acclimation. This finding suggests that conservatively maintaining a longer period of protection against larger-scale human disturbances beyond the 42-day period of initial physiological acclimation may be warranted. Further, recent evidence from this system suggests behavioural (Pero, 2021) and social acclimation (Pero *et al*., in press) durations lag behind the physiological acclimation period we observed for the Missouri elk population. Trait-specific temporal variation in acclimation duration suggests consideration of multiple biomarkers when evaluating post-release management actions is appropriate.

Glucocorticoid physiology is complicated, and the interpretation of data relative to population health can be nuanced. For example, low GC or fGCM levels on their own do not necessarily signify healthy functioning (Romero and Beattie, 2021). However, our results indicate that with sufficient long-term monitoring and access to adequate baseline or reference levels, fGCMs serve as a useful noninvasive bioindicator for assessing physiological acclimation. Adequate long-term monitoring and project reporting remain an issue for animal translocation projects (Berger-Tal *et al.,* 2020; Resende *et al*., 2021). As wildlife restoration is a costly conservation practice (Weise *et al.,* 2014), being able to use resources most efficiently is crucial to continued successful implementation. Our study supports the use of fGCMs as an innovative and efficient monitoring method called for by translocation specialists (Berger-Tal *et al.,* 2020).

## Management implications

We used faecal glucocorticoid metabolites as a noninvasive bioindicator of physiological acclimation in the restored Missouri elk population. We identified a relatively fast physiological acclimation period for Missouri elk compared to other large mammals for which physiological acclimation data are available. As such, post-release management at the release site relative to resource availability and disturbance reduction may facilitate acclimation and reduce the period of time recently translocated populations are at risk of post-release effects. Species-specific differences in translocation sensitivity likely contribute to the duration of the acclimation period and the period of time post-release management actions may be necessary. Increased resolution of number of species with known acclimation durations may thus contribute to improving the efficacy and efficiency of species-specific translocation guidelines and post-release management protocols.

## Funding

This work was supported by a U.S. Fish and Wildlife Service Wildlife Restoration Grant, the MDC, University of Missouri, University of Montana scholarship and fellowship (to E.P.), Rocky Mountain Elk Foundation and the Boone and Crockett Club University Program.

## Author contributions

E.P., C.C. and J.M. conceived and designed the research. E.P., C.C., A.H. and B.K. collected field data. R.M. and E.P. performed laboratory work. E.P. analysed the data. E.P., C.B., C.C., J.I. and J.M. wrote and edited the manuscript. C.B., A.H., B.K., J.S., L.H., J.I. and J.M. contributed materials/tools.

## Data Availability Statement

The data underlying this article will be shared upon reasonable request to the corresponding author.
